# Pericyte-derived cells participate in optic nerve scar formation

**DOI:** 10.3389/fphys.2023.1151495

**Published:** 2023-04-18

**Authors:** Julia Preishuber-Pflügl, Daniela Mayr, Veronika Altinger, Susanne M. Brunner, Andreas Koller, Christian Runge, Anja-Maria Ladek, Markus Lenzhofer, Francisco J. Rivera, Herbert Tempfer, Ludwig Aigner, Herbert A. Reitsamer, Andrea Trost

**Affiliations:** ^1^ Research Program for Experimental Ophthalmology and Glaucoma Research, Department of Ophthalmology and Optometry, University Hospital of the Paracelsus Medical University, Salzburg, Austria; ^2^ Laboratory of Stem Cells and Neuroregeneration, Institute of Anatomy, Histology and Pathology, Faculty of Medicine, Universidad Austral de Chile, Valdivia, Chile; ^3^ Center for Interdisciplinary Studies on the Nervous System (CISNe), Universidad Austral de Chile, Valdivia, Chile; ^4^ Translational Regenerative Neurobiology Group, Molecular and Integrative Biosciences Research Program (MIBS), Faculty of Biological and Environmental Sciences, University of Helsinki, Helsinki, Finland; ^5^ Institute of Tendon and Bone Regeneration, Spinal Cord Injury and Tissue Regeneration Center, Paracelsus Medical University, Salzburg, Austria; ^6^ Institute of Molecular Regenerative Medicine, Spinal Cord Injury and Tissue Regeneration Center, Paracelsus Medical University, Salzburg, Austria; ^7^ Spinal Cord Injury and Tissue Regeneration Center, Salzburg, Austria; ^8^ Director of the Research Program for Experimental Ophthalmology and Glaucoma Research, Salzburg, Austria

**Keywords:** pericyte, inducible PDGFRβ-P2A-CreER^T2^-tdTomato lineage tracing reporter mouse, fibrotic cells, optic nerve crush, scar formation

## Abstract

**Introduction:** Pericytes (PCs) are specialized cells located abluminal of endothelial cells on capillaries, fulfilling numerous important functions. Their potential involvement in wound healing and scar formation is achieving increasing attention since years. Thus, many studies investigated the participation of PCs following brain and spinal cord (SC) injury, however, lacking in-depth analysis of lesioned optic nerve (ON) tissue. Further, due to the lack of a unique PC marker and uniform definition of PCs, contradicting results are published.

**Methods:** In the present study the inducible PDGFRβ-P2A-CreER^T2^-tdTomato lineage tracing reporter mouse was used to investigate the participation and trans-differentiation of endogenous PC-derived cells in an ON crush (ONC) injury model, analyzing five different post lesion time points up to 8 weeks post lesion.

**Results:** PC-specific labeling of the reporter was evaluated and confirmed in the unlesioned ON of the reporter mouse. After ONC, we detected PC-derived tdTomato^+^ cells in the lesion, whereof the majority is not associated with vascular structures. The number of PC-derived tdTomato^+^ cells within the lesion increased over time, accounting for 60–90% of all PDGFRβ^+^ cells in the lesion. The presence of PDGFRβ^+^tdTomato^-^ cells in the ON scar suggests the existence of fibrotic cell subpopulations of different origins.

**Discussion:** Our results clearly demonstrate the presence of non-vascular associated tdTomato^+^ cells in the lesion core, indicating the participation of PC-derived cells in fibrotic scar formation following ONC. Thus, these PC-derived cells represent promising target cells for therapeutic treatment strategies to modulate fibrotic scar formation to improve axonal regeneration.

## 1 Introduction

Pericytes (PCs) are specialized cells located abluminal of endothelial cells (ECs) on microvessels, fulfilling numerous important functions (Diaz-Flores et al., 2009). PCs are important regulators of the microvasculature; they participate in capillary blood flow regulation (Hamilton et al., 2010) and are a component of the blood–retinal/–brain barrier (BRB/BBB) (Bell et al., 2010), playing a key role in tissue homeostasis. In addition, the capacity of PCs to differentiate *in vitro* into mesenchymal cell types (e.g., osteoblasts, chondrocytes, and adipocytes) and also non-mesenchymal cells (e.g., neural and glial lineage) has been confirmed in a multitude of studies [reviewed in the study by Trost et al. (2016) and Trost et al. (2019)]. Furthermore, the contribution of *in vitro* differentiated PCs to tissue repair and regeneration after transplantation has been demonstrated in several injury models [reviewed in the study by Trost et al. (2016) and Trost et al. (2019)]. Due to the lack of a unique PC marker, distinct markers have been used to identify and study PCs, including, but not limited to, PDGFRβ, Cspg4 (also named NG2), and Desmin (Zhu et al., 2022). Consequently, diverse PC lineage tracing mouse models with different promoters are available to study the involvement of PCs in tissue repair and regeneration, including the PDGFRβ-CreER^T2^ (Sheikh et al., 2015; Park et al., 2017), the PDGFRβ-P2A-CreER^T2^ (Cuervo et al., 2017; Ivanova et al., 2021; Mayr et al., 2021), the Cspg4-CreER™ (Zhu et al., 2011; Hill et al., 2015; Bruckner et al., 2018; Mayr et al., 2021), the GLAST-CreERT^T2^ (Goritz et al., 2011), and the tbx18-CreERT (Guimaraes-Camboa et al., 2017) transgenic lines [reviewed in the study by Trost et al. (2019)]. Due to the heterogeneity of used promoters, inconsistent findings are reported regarding the involvement of PCs in fibrotic scar formation and tissue repair: the participation of type 1 PCs (NG2^+^/Nestin^−^) was described in scar formation after spinal cord injury (SCI) using constitutive Nestin-GFP/NG2-dsRed transgenic mice (Birbrair et al., 2014). In line with these findings, *in vivo* differentiation of endogenous PCs into osteogenic cells was demonstrated in bone fracture healing using the inducible NG2-CreER; Rosa26RtdTomato mouse (Supakul et al., 2019). On the contrary, Soderblom et al. (2013) concluded just a marginal contribution of NG2^+^ PCs to the fibrotic scar after SCI using the inducible NG2-CreER™-tdTomato model. Applying distinct promoters, the participation of PC (-subpopulations) in scar formation was confirmed after SCI using the inducible Glast-CreER transgenic mouse (Goritz et al., 2011) and further in two kidney fibrosis models, using an inducible FoxD1-CreER^T2^ mouse (Humphreys et al., 2010). Using the tbx18-CreER^T2^ lineage tracing model, Guimaraes-Camboa et al. (2017) excluded *in vivo* trans-differentiation of PCs in different lesions, whereas Pham et al. (2021) reported a pro-fibrotic response of heart and brain PCs after vascular injury in the same mouse model. Contradicting findings regarding the endogenous trans-differentiation potential of PCs may result from differences in the severity of the applied injury models (e.g., small versus large cortico-striatial stab lesion), as demonstrated by Dias et al. (2021).

As PCs are described and discussed to participate in wound healing, they represent promising target cells for therapeutic treatment strategies to modulate/reduce fibrotic scar formation and improve (axonal) regeneration. However, findings are inconsistent, as the definition and characterization of PCs are not uniform across studies and several constitutive and inducible transgenic mouse models have been used to study PC trans-differentiation capacity. Importantly, fibrosis in CNS and the participation of PCs have been studied mainly in spinal cord or brain injury (Goritz et al., 2011; Dias et al., 2021; Pham et al., 2021). The ONC injury mouse model is an important experimental model to mimic and study human diseases like traumatic optic neuropathy or ON degeneration in glaucoma. In the optic nerve (ON), also part of the CNS, however, just a single study investigated the origin of fibrotic cells in scar formation following ON crush (ONC) in a constitutive Col1a1^+^ reporter mouse, designed to target fibroblasts (Liu et al., 2021). Therefore, we investigated the participation and trans-differentiation of endogenous PC-derived cells in an ONC injury model, analyzing five different post-lesion time points up to 8 weeks post-lesion (wpl), using the inducible PDGFRβ-P2A-CreER^T2^-tdTomato lineage tracing mouse.

## 2 Materials and methods

### 2.1 Experimental animals

All animal procedures were conducted in accordance with the ARVO Statement for the Use of Animals in Ophthalmic and Vision Research and were approved by the national and institutional animal care and use committee (20901-TVG/125/7-2018). Mice were housed in the animal facility at the Department of Ophthalmology of the University Hospital of the Paracelsus Medical University, Salzburg, Austria, under environmentally controlled conditions with a 12-hour light/dark cycle and *ad libitum* access to standard rodent chow and water. The homozygous PDGFRβ-P2A-CreER^T2^ mouse (Stock #030201, Jackson Laboratory) (Cuervo et al., 2017) was crossbred with the homozygous B6. Cg-Gt (ROSA)^26Sortm9(CAG-tdTomato)Hze^ reporter mouse (stock #007909, Jackson Laboratory) (Madisen et al., 2010). Double transgenic, heterozygous male and female PDGFRβ-P2A-CreER^T2^-tdTomato mice (termed PDGFRβ-tdTomato in the following) were used to study the fate of PC-derived cells following ONC.

### 2.2 Reporter gene activation by TAM administration

Tamoxifen (TAM) was administered by subcutaneous (s.c.) injections (BD Micro-Fine 0.5 mL; 29G; U-100 Insulin Syringe; REF 324824) up to three times between postnatal (P) days 4 and 14 with a maximum of one dose per day. Mice were induced with 100 mg/kg body weight TAM (Sigma-Aldrich; T5648) dissolved in corn oil (Sigma-Aldrich; C8267). As negative controls, corn oil-induced PDGFRβ-P2A-CreER^T2^-tdTomato mice were used.

### 2.3 Optic nerve crush

Male and female TAM-induced PDGFRβ-P2A-CreER^T2^-tdTomato mice (∼12 weeks of age, *n* = 34) were subjected to a unilateral ONC of the right eye, leaving the dura intact, without prolonged ischemia to the entire ocular vasculature. The animals were anesthetized with a combination of ketamine hydrochloride/xylazine (60/3 mg/kg i.p. Sigma-Aldrich, Vienna, Austria). The treated eye received topical anesthesia with 4.0 mg oxybuprocain hydrochloride (0.4% Novain, Agepha Pharma, Senec, Slovakia) and was disinfected with a povidone–iodine complex solution (5% Betaisodona solution, Salzburger Landesapotheke, Salzburg, Austria). For the ONC, the ON was surgically uncovered using a surgical microscope (Universal S3B + OPMIMD, Zeiss West, Göttingen, Germany) by making a small incision in the temporal/superior conjunctiva. Following this first incision, blunt dissection was performed to avoid tissue damage and intraorbital trauma. The exposed ON was grasped and crushed 1–3 mm behind the ON head for 10 s (Dumont tweezer style 7, 0102-7-PO, Switzerland). After the ONC, retinal blood circulation was monitored by applying a cover slip on the cornea and observing retinal blood circulation with the surgical microscope. Prior to the application of the cover slip, the eye was moisturized (Hylo-COMOD, sodium hyaluronate 1 mg/mL, Ursapharm, Saarbrücken, Germany). During the whole procedure (5–10 min), the animals were placed on a heat blanket to maintain normal body temperature (37°C–38°C, Homeothermic Blanket Control Unit, Harvard Apparatus, Holliston, MA, United States). To prevent possible infections, Dexagenta-POS eye ointment (0.3 mg Dexamethasone, 5.0 mg Gentamicin Sulfate, Ursapharm, Saarbrücken, Germany) was applied on the treated eye; the contralateral untreated eye received Vita-POS eye ointment (250 I.E.,/g Retinol palmitate, Ursapharm) to prevent desiccation during the wake-up period.

### 2.4 Tissue preparation

The animals were sacrificed 2 days (*n* = 3/3 female/male), 4 days (*n* = 3/2), 1 week (*n* = 6/4), 3 weeks (*n* = 3/3), and 8 weeks (*n* = 4/3) after ONC by an overdose of pentobarbital i.p. (Release, 300 mg/mL; 600 mg/kg, WDT, Garbsen, Germany). Eye cups, including ONs, were removed and fixed in 4% paraformaldehyde for 1 h at room temperature (RT), rinsed in 0.1 M phosphate buffer (PO_4_, pH 7.4) overnight (4°C), and transferred into PO_4_ containing 15% sucrose (24 h at 4°C). Eyes were embedded in tissue embedding medium (NEG50, Fisher Scientific, Vienna, Austria) and frozen at −80°C using liquid nitrogen-cooled isopentane (VWR, Austria) and stored at −20°C until further processing. The unlesioned, contralateral ON served as the control.

### 2.5 Immunofluorescence

ONs were cut longitudinally using a cryostat (HM 550, Microm, Histochom, Wiener Neudorf, Austria), and serial sections of 12 µm were applied to six adhesion slides (Superfrost Plus, Thermo Fisher, Vienna, Austria), each slide containing every 6th section (72 µm distance between each section on the slide), including sections from the edge and center of each ON. Sections were air-dried overnight at RT and stored at −80°C until immunofluorescence (IF) was performed. Antigen retrieval was performed prior to IF with 10 mM citrate buffer (pH 6.0; Sigma-Aldrich) at 65°C for 1 h, followed by three washing steps in 0.05 M TBS (each 5 min). The IF procedure we performed is the same as that described in the study of Mayr et al. (2021). Primary antibodies used are listed in Table 1. Negative controls were performed by omission of the primary antibodies during incubation and resulted in the absence of immunoreactivity (IR). After IF labeling, the lesion site was identified by the absence of cells at the early time points after the lesion; from 1 wpl onward, the lesion site was clearly distinguishable from healthy ON tissues by its hypercellularity (DAPI). For the analysis, 2–3 hypercellular regions (=lesion) were recorded per optic nerve, and the average values were included in the statistical analysis.

**TABLE 1:** Primary antibodies used in immunofluorescence.

Protein	Host	Company	Catalog no.	Dilution	Cell/structures
PDGFRβ	Goat	R&D	AF1042	1:150	Pericytes (PCs) and vSMCs
NG2	Rabbit	Millipore	AB5320	1:300	PCs and vSMCs, oligodendrocyte precursor cells (OPCs)
CD31	Rabbit	Thermo Scientific	PA5-16301	1:50	Endothelial cells (ECs)
Iba1	Rabbit	Wako	019-19741	1:500	Microglial cells
Iba1	Goat	Abcam	ab107159	1:500	Microglial cells
GFAP	Guinea pig	Progen	GP52	1:500	Astrocytes
Sox10	Goat	Santa Cruz	sc-17342	1:50	OPCs and premyelinating oligodendrocytes
Olig2	Rabbit	Millipore	AB9610	1:250	OPCs and premyelinating oligodendrocytes
Desmin	Rabbit	Abcam	ab15200	1:100	Pericytes and vSMCs
MBP	Rat	Bio-Rad	MCA409S	1:200	Mature myelinated oligodendrocytes
PLP	Rabbit	Santa Cruz	Sc-98781	1:100	Mature myelinated oligodendrocytes
Col1a1	Rabbit	Abcam	ab21286	1:150	Fibroblasts
Col1a1	Rabbit	Abcam	ab34710	1:300	Fibroblasts

### 2.6 Documentation

For documentation, a confocal laser-scanning unit (Axio Observer Z1 attached to LSM710, Zeiss, Göttingen, Germany; ×20 dry and ×40 oil immersion objective lenses, with numeric apertures 0.8 and 1.30, respectively; Zeiss) was used. Sections were imaged using the appropriate filter settings for AF488 (495 nm excitation), tdTomato (554 nm excitation), AF647 (650 nm excitation), and DAPI (345 nm excitation). Up to four channels were detected consecutively and merged. For imaging and documentation of whole optic nerve sections, the Slide Scanner (VS-120-L Olympus slide scanner 100-W system) was used, and images were processed using the Olympus VS-ASW-L100 program (Olympus, Shinjuku, Tokyo, Japan).

### 2.7 Cell counting and polarization analysis

To characterize tdTomato reporter expression in unlesioned ONs, tdTomato^+^ cells were analyzed in whole longitudinal optic nerve sections documented by Slide Scanner. The number of vascular and non-vascular associated tdTomato^+^ cells was analyzed with respect to PDGFRβ [PC/vascular smooth muscle cells (vSMCs)] and CD31 (endothelial cells, ECs) labeling in longitudinal ON sections at three TAM induction time points and calculated per ON area (mm^2^). Following ONC, the lesion area was defined by the hypercellular tdTomato^+^ region, and tdTomato^+^ cells were calculated with respect to DAPI^+^ cell nuclei, their co-localization with PDGFRβ^+^ cells, their co-localization with Ki67, and their association with CD31 vascular structures at 1 wpl, 3 wpl, and 8 wpl. Area definition, cell count, and co-localization analysis within the lesion were performed using the analysis tools of ZEN 2.6 lite (Zeiss). Collagen deposition was detected by applying a polarization filter relying on the birefringence of collagen fibers (LSM710, AxioVision, Zeiss). The mean polarization signal intensity was compared between the lesioned area (= hypercellular region), the proximal (defined in direction to optic nerve head (ONH)) region, and the distal (defined in direction to chiasma) region of the lesion, set in relation to the proximal intensity values (ZEN 2.6 lite). All analyses included up to three distinct ON sections per animal (representing different planes within the lesioned ON), and the mean values were included in the calculation of the group. Statistical analysis was performed using GraphPad Prism 9 Software (GraphPad, California, United States), applying appropriate statistical tests as indicated. *p*-values ≤0.05 were considered statistically significant.

## 3 Results

### 3.1 TdTomato reporter expression in PCs in the unlesioned ON

**FIGURE 1:**
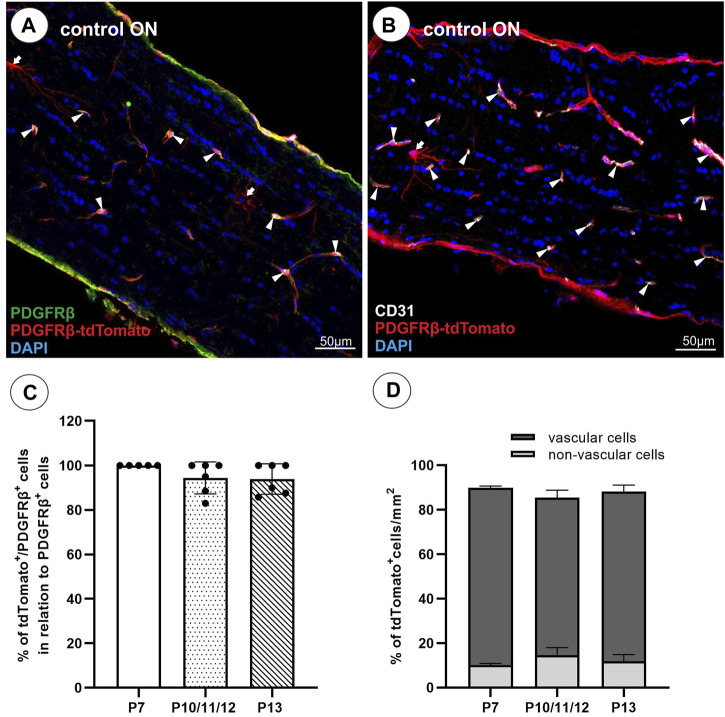
TdTomato reporter expression in the unlesioned ON. **(A)** Almost all PDGFRβ^+^ (green) cells revealed tdTomato^+^ expression (red). **(B)** The majority of tdTomato^+^ cells are associated with CD31^+^ vascular structures (white), and a minority of tdTomato^+^ cells revealed a branched, non-vascular morphology. **(C)** The amount of tdTomato^+^/PDGFRβ^+^ cells in the healthy ON calculated in relation to all PDGFRβ^+^ cells (%) following a TAM induction at P7, P10/11/12, or P13 (n = 5–6 per induction procedure). **(D)** Comparison of the percentage of tdTomato^+^ cells associated with vascular structures versus non-vascular structures following a TAM induction at P7 (*n* = 2), P10/11/12 (*n* = 2), and P13 (*n* = 3). Filled arrowheads show vascular tdTomato^+^ cells, filled arrows show non-vascular tdTomato^+^ cells.

TdTomato reporter expression of the PDGFRβ-tdTomato lineage tracing mouse has been described in the retina in PCs, vascular smooth muscle cells (vSMCs), and Müller glia cells upon TAM induction at different postnatal time points (Mayr et al., 2021). However, as a characterization of the model is missing for the ON, tdTomato expression was analyzed in contralateral unlesioned ONs before studying ON lesions. In the present study, TAM induction of the PDGFRβ-tdTomato reporter mice was performed up to three times between postnatal days 4 and 14. Association of tdTomato^+^ cells to vascular structures was analyzed using the PC/vSMC marker PDGFRβ (Figure 1A) and the EC marker CD31 (Figure 1B) in the unlesioned control ON. Three different time points of TAM induction (P7; P10/11/12; P13 per induction protocol) were analyzed at 12 weeks of age, and tdTomato^+^ cells showing a nucleus were counted in the analyzed longitudinal ON section. Between 93.9% ± 6.9% (P13) and 100% (P7) of PDGFRβ^+^ PCs were labeled by tdTomato following TAM induction (Figure 1C). Following the three different TAM induction procedures, the portion of tdTomato^+^ cells revealing association with CD31^+^ vascular structures remained constant with a total average number of 60.2 ± 3.8 tdTomato^+^ cells/mm^2^ (87.8% ± 2.3% on average, Figure 1D). Of note, corn oil-induced PDGFRβ-tdTomato reporter mice revealed 4.9 ± 2.7 tdTomato^+^ cells/mm^2^ (*n* = 4) in total. No labeling was detected in TAM-induced tdTomato mice.

**FIGURE 2:**
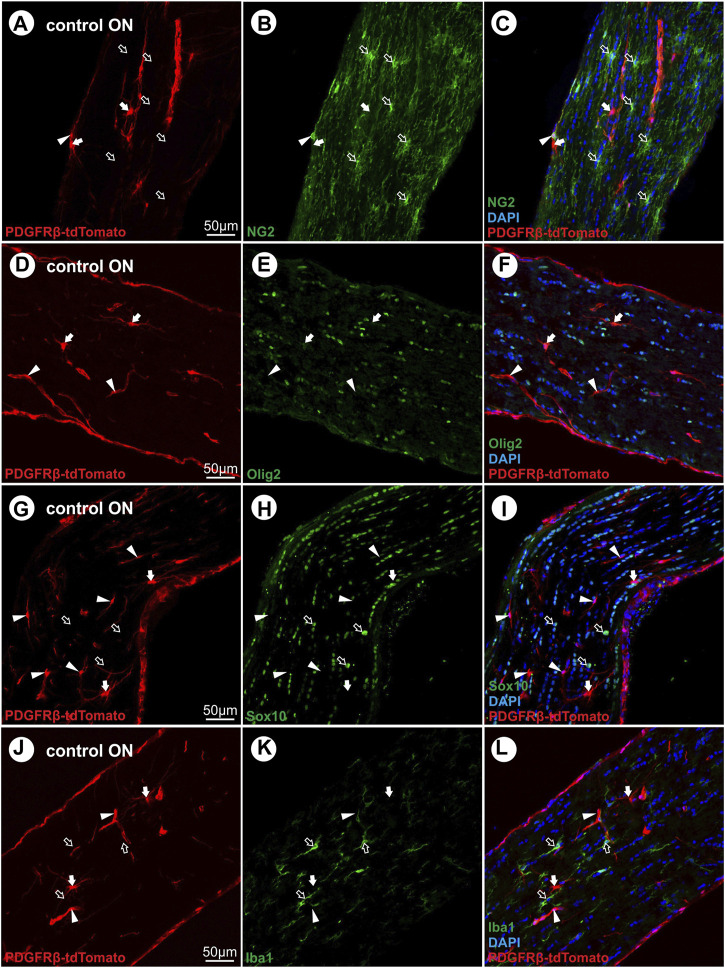
Characterization of non-vascular branched PDGFRβ-tdTomato^**+**^ cells with oligodendroglial (NG2, Olig2, Sox10) and microglial (Iba1) markers. **(A–C)** Non-vascular branched tdTomato^+^ cells (red) revealed no co-localization with NG2 (green), an oligodendroglial as well as PC marker. **(D–F)** No co-localization was observed for non-vascular and vascular tdTomato^+^ cells with Olig2 (green). **(G–I)** The non-vascular tdTomato^+^ cells further lacked Sox10-IR (green) and **(J–L)** revealed no co-localization with the microglial marker Iba1 (green). Filled arrows show non-vascular tdTomato^+^ cells, filled arrowheads show vascular tdTomato^+^ cells, and blank arrows show cells with IR for oligodendroglial or microglial markers.

Similar to the retina, in the ON of the PDGFRβ-tdTomato lineage tracing mouse, some non-vascular associated cells are labeled in addition to vascular cells. To further characterize these non-vascular associated branching tdTomato^+^PDGFRβ^−^ cells in the unlesioned ON, which account for approximately 12% of the total tdTomato^+^ cell population (Figure 1D), co-localization was studied with markers detecting oligodendrocyte precursor cells (OPCs; Sox10, Olig2, NG2), premyelinating oligodendrocytes (Olig2, Sox10), mature myelinated oligodendrocytes (MBP, PLP), and microglial cells (Iba1). NG2, a marker for perivascular cells and also OPCs, revealed no co-localization with non-vascular associated branching tdTomato^+^ cells but only with vascular tdTomato^+^ cells (Figures 2A–C). Furthermore, non-vascular associated branched tdTomato^+^ cells revealed the absence of Olig2-IR (Figures 2D–F), Sox10-IR (Figures 2G–I), MBP-IR (data not shown), PLP-IR (data not shown), or Iba1-IR (Figures 2J–L).

### 3.2 PC-derived tdTomato^+^ cells contribute to scar formation following ONC

To evaluate the contribution of tdTomato^+^ cells to scar formation and wound healing following ONC, the presence of tdTomato^+^ cells within the lesion was analyzed at five different time points post ONC (2 days post lesion (dpl), 4 dpl, 1 wpl, 3 wpl, and 8 wpl).

**FIGURE 3:**
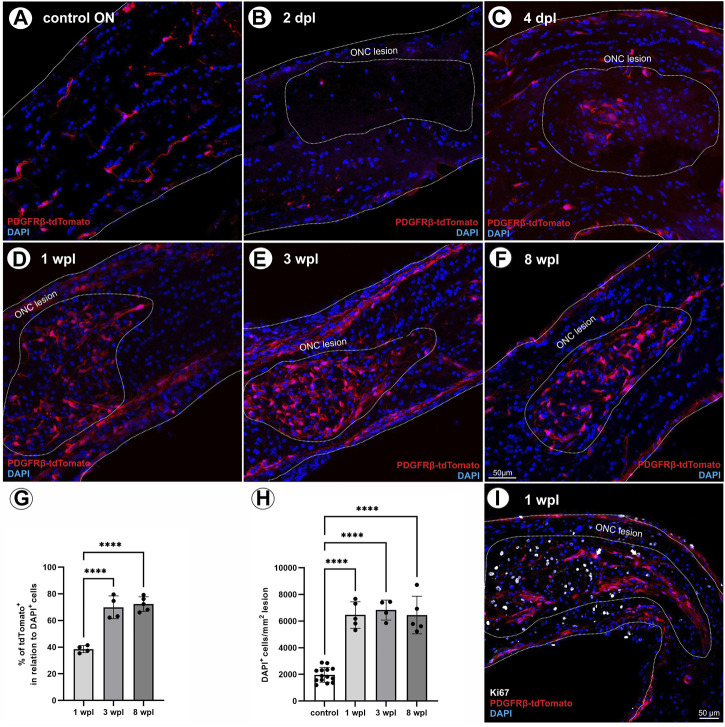
Localization of tdTomato+ cells in the lesion area over time. **(A)** In control ONs, tdTomato^+^ cells (red) are mainly associated to vascular structures. **(B)** At 2 dpl, a nearly cell-free region is detected at the site of ONC. **(C)** At 4 dpl, a small hypercellular region is visible within the lesion, including also tdTomato^+^ cells (*n* = 5). **(D)** At 1 wpl (*n* = 9), **(E)** 3 wpl (*n* = 4), and **(F)** 8 wpl (*n* = 7), tdTomato^+^ cells were detected evenly distributed in the lesion area. **(G)** The number of tdTomato^+^ cells in relation to DAPI^+^ cells within the lesion. **(H)** The number of DAPI^+^ cells in control ONs in comparison to DAPI^+^ cells within the lesioned area at 1 wpl, 3 wpl, and 8 wpl. **(I)** At 1 wpl, the proliferation marker Ki67 is detected within the lesion, revealing just sporadic co-localization with TdTomato^+^ cells (filled arrows, n = 3). **(A–F)** Representative images; the ONC lesion is marked by the dotted line. Significance was calculated by one-way ANOVA and Tukey’s multiple-comparison test; *p < 0.05, **p < 0.01, ***p < 0.001, and ****p < 0.0001.

In the unlesioned control ON, tdTomato^+^ cells are associated with vascular structures (Figure 3A). Analyzing tdTomato^+^ cells within the lesion at 2 dpl, single tdTomato^+^ cells were detected in one of six lesioned ONs, surrounded by an almost cell-free region (Figure 3B). At 4 dpl, repopulation of cells to the lesion site was observed, detecting a small hypercellular region, showing several cells expressing the tdTomato reporter (Figure 3C). At 1 wpl, the number of tdTomato^+^ cells within the lesion increased and represented 38.5% ± 2.7% of the total number of DAPI^+^ cells within the lesion (Figures 3D, G). At 3 wpl and 8 wpl, 69.9 ± 8,5% and 72.4% ± 5.5% of DAPI^+^ cells within the lesion were tdTomato^+^, showing a significant increase in comparison to 1 wpl (1 vs. 3 wpl: *p* < 0.0001; 1 vs. 8 wpl: *p* < 0.0001; Figures 3E–G). Analyzing the total number of DAPI^+^ cells per mm^2^ ON, the unlesioned contralateral ON revealed a cell density of 1966 ± 554.6 DAPI^+^ cells/mm^2^. Post lesion, a significant increase in cell density was detected in the lesion area at 1 wpl (6,462 ± 995.4 DAPI^+^ cells/mm^2^; *p* < 0.0001) and remained constantly high at 3 wpl (6,830 ± 751.3 DAPI^+^ cells/mm^2^; *p* < 0.0001) and 8 wpl (6,457 ± 1415 DAPI^+^ cells/mm^2^, *p* < 0.0001; Figure 3H).

To investigate a potential proliferative state of tdTomato^+^ cells within the lesion, the expression of Ki67, a specific marker for cell proliferation, was analyzed 1 wpl and revealed that 19.3% ± 2.9% of the DAPI^+^ cells are Ki67^+^. However, only sporadic tdTomato^+^ cells were positive for Ki67, accounting for 1.5% ± 0.4% of the Ki67^+^ cell population (Figure 3I).

### 3.3 TdTomato^+^ cells in the lesion reveal reduced association with the vasculature but co-localization with PDGFRβ^+^ network-like structures

**FIGURE 4:**
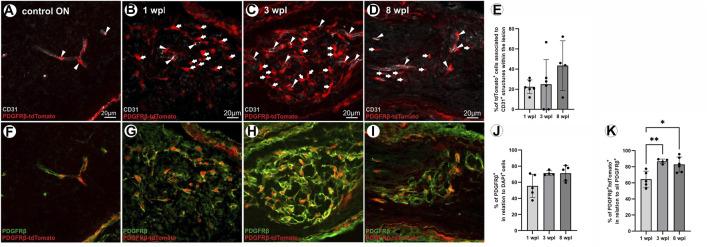
Vascular association of tdTomato^+^ cells and PDGFRβ IR of tdTomato^+^ cells within the lesion over time. **(A)** In the control ON, the majority of tdTomato^+^ cells are associated with CD31^+^ ECs, indicating vascular structures. **(B)** At 1 wpl, tdTomato^+^ cells are homogenously distributed within the lesion, showing low association with CD31^+^ vascular structures. **(C)** At 3 wpl and **(D)** 8 wpl, again, minor vascular association of tdTomato^+^ cells was detected within the lesion. **(E)** The % of association of tdTomato^+^ cells with CD31^+^ vascular structures in the lesion, at the three time points investigated (*n* = 4–6). **(F)** In the control ON, PDGFRβ is specifically expressed in vascular cells. Within the lesion, however, **(G–I)** PDGFRβ^+^ cells form a network-like structure, mainly lacking association with vascular structures. **(J)** The population of PDGFRβ^+^ cells within the lesion 1 wpl (*n* = 5), 3 wpl (*n* = 4), and 8 wpl (*n* = 5). **(K)** The percentage of these PDGFRβ^+^ cells that are also positive for tdTomato is presented for 1 wpl (*n* = 5), 3 wpl (*n* = 4), and 8 wpl (*n* = 6). Significance was calculated by one-way ANOVA and Tukey’s multiple comparisons test; *p < 0.05 and **p < 0.01. All pictures are representative pictures. Filled arrowheads show vascular tdTomato^+^ cells, and filled arrows show tdTomato^+^ cells not associated with vasculature.

As in the control ON, the majority of tdTomato-expressing cells is associated to CD31^+^ ECs (Figure 4A), and the association of tdTomato^+^ cells to the vasculature within the lesion was studied following ONC. At 4 dpl, no vascular structures were detected within the lesion area (data not shown). At 1 wpl, 22.4% ± 6.9% of tdTomato^+^ cells within the lesion were associated with vascular structures (Figures 4B, E). At 3 wpl, 24.9% ± 24.6% of tdTomato^+^ cells in the lesion revealed an association with CD31^+^ vascular structures (*p* > 0.05, Figures 4C, E), which slightly increased to 43.4% ± 24.8% at 8 wpl (1/3 vs. 8 wpl: *p* > 0.05, Figures 4D, E).

In the unlesioned ON, the PC marker PDGFRβ is associated with CD31^+^ vascular structures (Figure 4F). Following ONC, a PDGFRβ^+^ network is detected within the lesion and the majority of cells therein are not associated with vascular structures. Thus, the extent and density of PDGFRβ^+^ cells and the co-localization of PDGFRβ^+^ cells with tdTomato-IR were analyzed in the lesion. In total, 1 wpl 55.6% ± 14.3% of DAPI^+^ cells in the lesion are PDGFRβ^+^ (Figures 4G, J). The amount of PDGFRβ^+^ cells within the lesion increased further to 71.4% ± 3.1% at 3 wpl (1 wpl vs. 3 wpl, *p* = 0.107, Figures 4H, J) and to 71.3% ± 9.6% at 8 wpl (1 wpl vs. 8 wpl, *p* = 0.088, Figures 4I, J). Analyzing the number of tdTomato^+^ cells in relation to the PDGFRβ^+^ cell population in the lesion, at 1 wpl, 64.5% ± 10.1% are PDGFRβ^+^tdTomato^+^, which significantly increased to 86.8% ± 2.8% at 3 wpl (*p* = 0.005) and 82.83% ± 9.34% at 8 wpl (1 wpl vs. 8 wpl, *p* = 0.010, Figure 4K). A small proportion of the PDGFRβ^+^ cell population was negative for tdTomato.

### 3.4 Expression of the microglial marker Iba1 and the absence of GFAP + filaments in the lesion over time

**FIGURE 5:**
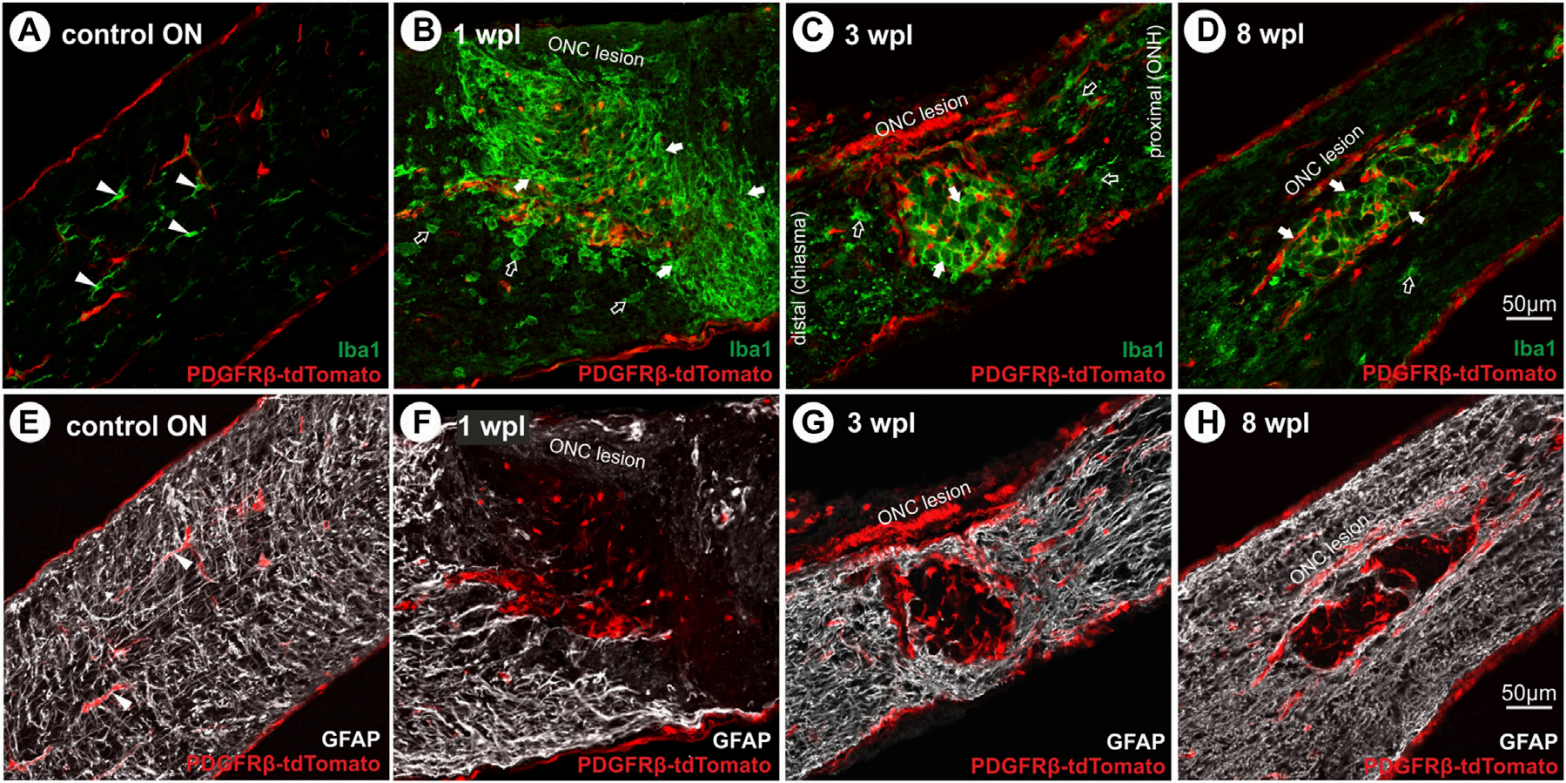
Microglial cell activation and absence of GFAP^+^ filaments in the lesion over time. **(A)** In the control ON, Iba1^+^ microglial cells (green) display a ramified form with motile processes, lacking tdTomato expression. Following ONC, microglial cells become activated and reveal an amoeboid form organized in a grit-like pattern at **(B)** 1 wpl, **(C)** 3 wpl, and **(D)** 8 wpl within the lesioned area (green). Furthermore, activated cells are detected distal and proximal to the lesion. **(E)** In the control ON, GFAP^+^ filaments of astrocytes are homogenously distributed (white), interacting with vascular cells *via* their end feet. Following ONC, the lesion area is devoid of GFAP^+^ filaments at **(F)** 1 wpl and **(G)** 3 wpl. Single repopulating GFAP^+^ filaments can be detected **(H)** 8 wpl. Filled arrowheads represent microglial or astroglial cells, filled arrows mark activated microglial cells in the lesion, and open arrows mark those outside the lesion.

To further characterize tdTomato^+^ cells in the ONC lesion, co-localization with microglial (Iba1) and astrocytic (GFAP) markers was tested. In the unlesioned ON, Iba1^+^ microglial cells were in a monitoring state, displaying a ramified morphology with motile processes (Figure 5A) and showing no tdTomato expression. Within the lesion, they revealed an activated amoeboid form organized in a grid-like pattern at 1 wpl, 3 wpl, and 8 wpl (Figures 5B–D). Although co-localization of Iba1 and tdTomato-IR was absent in nuclei with strong tdTomato expression, a faint co-localization is detected in cellular compartments with low tdTomato expression. Activated microglial cells with an amoeboid form were also present proximal and distal to the lesion, though more loosely arranged (Figures 5B–D). To analyze active PDGFRβ expression 8 wpl, antibody-based detection was performed, which revealed a close proximity of Iba1 and PDGFRβ-IR at the cell margin/boundary (Supplementary Figure S1). However, a clear co-localization or assignment to the same cell cannot be made with the methods used here and must be investigated in more detail in future studies. Investigating the proliferative state of Iba1^+^ cells within the lesion 1 wpl, 92.5% ± 0.8% of Ki67^+^ cells showed co-localization with Iba1 (data not shown). However, this Iba1^+^Ki67^+^ population represents a small subpopulation of Iba1^+^ cells in the lesion at 1 wpl.

Furthermore, GFAP^+^ filaments of astrocytes were homogenously distributed in the unlesioned ON, interacting with vascular cells *via* their end feet (Figure 5E), lacking tdTomato expression. Following ONC, the lesion was almost devoid of GFAP^+^ filaments at all time points investigated (Figures 5F–H). They formed a glial scar confining the lesion, showing no co-localization with tdTomato^+^ cells.

### 3.5 Collagen deposition within the lesioned ON

**FIGURE 6:**
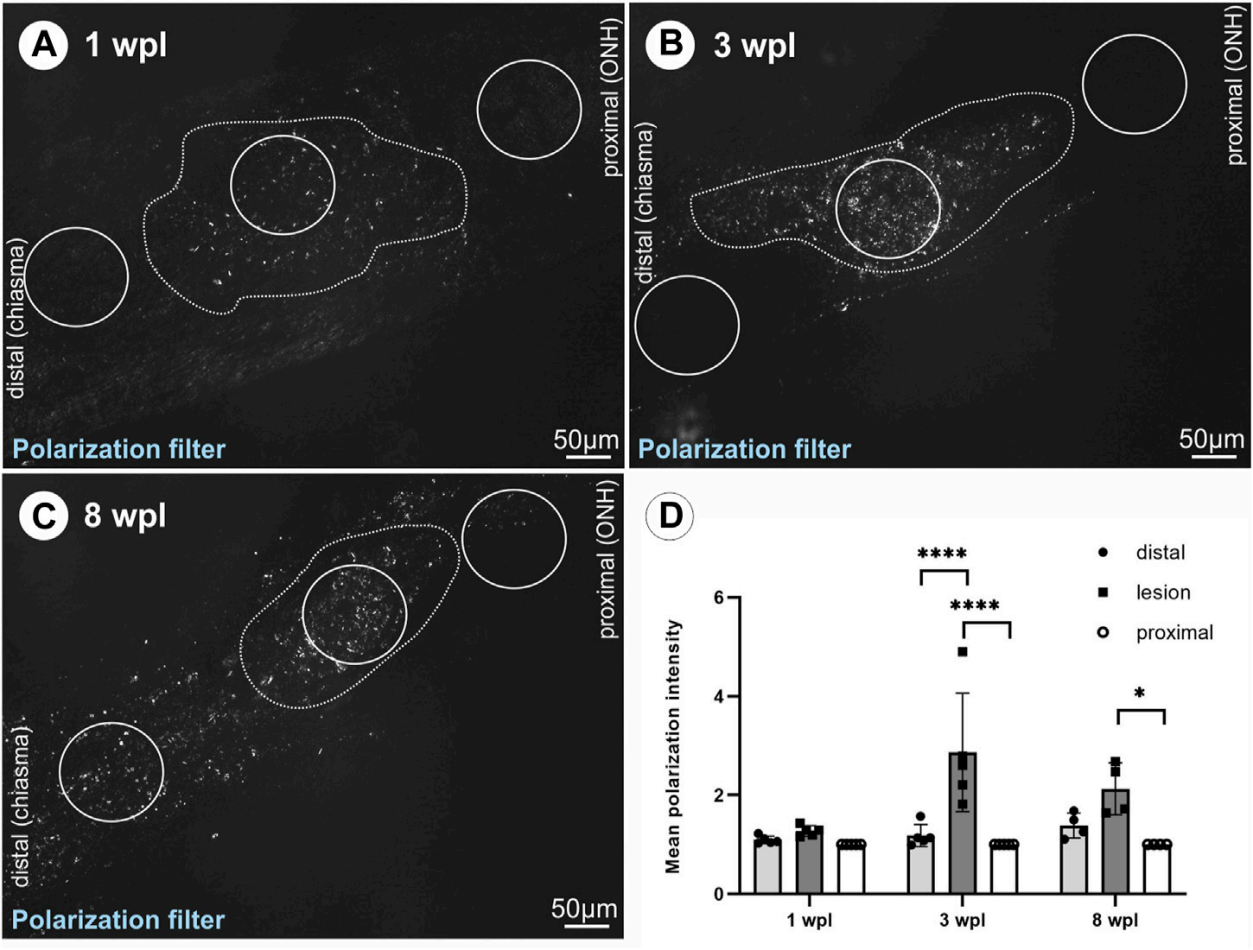
Detection of collagen deposition *via* polarization signal in the lesioned ON. The polarization signal within the lesioned area versus distal or proximal areas of the ON tissue is shown for **(A)** 1 wpl (*n* = 5), **(B)** 3 wpl (*n* = 5), and **(C)** 8 wpl (*n* = 4). **(D)** Graph showing mean polarization intensity comparing lesioned, distal, and proximal ON tissue at 1 wpl, 3wpl, and 8 wpl. The dotted line highlights the hypercellular lesioned area. Significance was calculated by two-way ANOVA and Šìdàk’s multiple-comparison test; *p < 0.05 and ***p < 0.001.

As fibrotic scars are composed of collagen and several studies describe PCs or perivascular cells as collagen-producing cells in CNS lesions with intact meninges, collagen 1a1 deposition was studied following ONC. As the use of two different collagen 1a1 antibodies did not result in specific labeling in ON tissue, polarization filter analysis was performed to detect collagen deposition, relying on the birefringence of collagen fibers. At 1 wpl, no difference in the mean polarization intensity was detected between the lesioned, distal, and proximal ON areas (Figures 6A, D). At 3 wpl, a significant increase in polarization intensity was detected comparing the proximal vs. lesioned areas and the lesioned vs. distal areas (*p* < 0.0001, Figures 6B, D), showing that the polarization signal is limited to the lesioned area. At 8 wpl, polarization signals were also detected distal to the lesion (Figure 6C), resulting in no significant difference in mean polarization intensity between the distal area and lesion. Although polarization signals were also detected in proximal areas, but to a lesser extent, a significant difference remained in comparison to the lesioned area (*p* = 0.014, Figures 6C, D).

## 4 Discussion

The potential involvement and modulation of PCs in wound healing and tissue regeneration have been achieving increasing attention for years. Although diverse studies have analyzed the participation of PCs in the spinal cord and brain scar tissue, a thorough investigation of PCs in the lesioned ON is missing. Using the inducible PDGFRβ-P2A-CreER^T2^-tdTomato lineage tracing mouse, the participation of PC-derived cells in the fibrotic scar of the lesioned ON was demonstrated for the first time. We observed that the number of PC-derived tdTomato^+^ cells increased in the lesion area with time, accounting for 60%–90% of all PDGFRβ^+^ cells in the lesion. The majority of these cells lacked vascular association, indicating a distinct function in the fibrotic scar. Importantly, the simultaneous presence of PDGFRβ^+^tdTomato^-^ cells in the lesion indicates that fibrotic cells with different origins exist within the ON scar, indicating the possible existence and participation of diverse PC subpopulations in ON wound healing.

### 4.1 Characterization of tdTomato reporter expression in unlesioned ON cells

In the present study, the inducible PDGFRβ-P2A-CreER^T2^-tdTomato mouse model was used to target PCs. However, as the PDGFRβ promoter is not exclusively expressed in PCs, a thorough analysis of the tdTomato reporter-positive cells was performed in the contralateral unlesioned ON upon TAM induction. To identify PCs in the unlesioned ON, the markers PDGFRβ, NG2, and desmin were used. Although we and other researchers observed that NG2 and desmin specifically label PCs and vSMCs in retinal tissue (Hughes and Chan-Ling, 2004; Bruckner et al., 2018), in the ON, oligodendroglial cells and astrocytes were additionally targeted, respectively (data not shown). Therefore, these markers were not further used to define PCs in this study. PDGFRβ represents an established marker to identify PCs; however, PDGFRβ is also expressed in vSMCS and perivascular fibroblasts (Vanlandewijck et al., 2018). To solve this problem and to discriminate PCs from vSMCs and perivascular fibroblasts, they were identified not only by PDGFRβ-IR but also by their association with CD31^+^ ECs in combination with their capillary localization in the present study. This combination was then further used to characterize tdTomato reporter labeling in the unlesioned ON. Furthermore, induction time points with TAM were chosen at early postnatal time points (P4–P14) to target PCs involved in the development of the retinal capillary plexus (Sapieha, 2012). With regard to these criteria, we observed that 90% of tdTomato^+^ cells co-expressed PDGFRβ and were associated with CD31^+^ capillary structures in the unlesioned ON, indicating that the PDGFRβ-P2A-CreERT2-tdTomato mouse model indeed enables lineage tracing of PC-derived cells in the ON. Nevertheless, as long as the lack of reliable (exclusive) PC markers persists, their clear discrimination from vSMCs and perivascular fibroblasts is hampered (Dorrier et al., 2022) and aggravates the assignment of distinct roles to the specific cell types. Consequently, in the past, diverse cells may have been mistakenly defined as PCs, and thus, conclusions were drawn on the function of PCs, which may not be specific for PCs. However, by performing single-cell RNA sequencing in four different types of muscle tissue, a recent study identified molecular signatures to distinguish fibroblasts and mural cells (Muhl et al., 2020), providing additional markers for future discrimination.

Interestingly, we also observed single PDGFRβ^+^ cells associated with the vasculature, which were tdTomato negative. These cells potentially represent PCs with an inactive PDGFRβ promoter at the time point of TAM induction, indicating the presence of various PC subpopulations with distinct expression profiles. This is in line with studies suggesting PC heterogeneity and organotypicity (Vanlandewijck et al., 2018; Muhl et al., 2020) or the existence of different PC subpopulations (Goritz et al., 2011; Birbrair et al., 2013; Yang et al., 2022). In addition to capillary tdTomato^+^ cells, approximately 12% of tdTomato^+^ cells revealed a branched morphology, absence of vascular association, and lack of co-expression with PDGFRβ or the oligodendroglial lineage markers NG2, Olig2, and Sox10. Whether these branched tdTomato^+^ cells represent a yet unknown PC subpopulation, cells that transdifferentiated from PCs, or are a specific subtype of oligodendroglial cells with an active PDGFRβ promoter at early developmental time points needs to be addressed in future studies.

### 4.2 PC-derived tdTomato^+^ cells contribute to fibrotic scar formation

Although many studies investigated the glial scar, axonal regrowth, and regeneration following ON lesion, only few studies focused on unveiling the cellular components of the fibrotic scar. Scars in the CNS are formed by an outer glial scar, forming an injury border, and an inner fibrotic scar at the core of the injury, revealing a high density of PDGFRβ^+^ cells in ON (Trost et al., 2017; Liu et al., 2021) and CNS injury models (Goritz et al., 2011; Soderblom et al., 2013; Birbrair et al., 2014). The characterization of these PDGFRβ^+^ cells is contradicting as they were, e.g., defined as fibroblasts distinct from NG2^+^ PCs (Birbrair et al., 2014) or characterized as stromal cells derived from a PC subpopulation (Goritz et al., 2011). In the present study, we demonstrated a PC-derived origin for a large subpopulation of PDGFRβ^+^ cells in the lesion by their concomitant expression of the reporter tdTomato. Additionally, we detected PDGFRβ^+^tdTomato^-^ cells in the lesion, indicating the presence of a distinct fibrotic cell subpopulation with a different origin compared to promoter-targeted PCs. These findings suggest a heterogeneous origin for the fibrotic cell (sub-) populations within the fibrotic scar. Whether these potential fibroblast subpopulations of different origins fulfill diverse functions remains to be elucidated. On the other hand, lack of tdTomato expression in the PDGFRβ^+^ subpopulation may also result from incomplete PC labeling at the time point of TAM induction.

Analyzing the contribution of PC-derived tdTomato^+^ cells to the scar formation following ONC, we detected that 38% of the cells in the lesion core revealed tdTomato expression at 1 wpl, which increased to 70% at 3 wpl and 8 wpl. The majority of these tdTomato^+^ cells revealed no vascular association, indicating a function distinct from vascular processes. In line, reduced vascular association in the lesion core was also demonstrated for a distinct PC subpopulation, defined as type A PCs, reported to participate in PC-derived fibrosis in a conserved mechanism across different CNS lesions models (Dias et al., 2021). Therefore, the presence of tdTomato^+^ cells in the lesion core that are not associated with vasculature indicates the participation of PC-derived cells in fibrotic scar formation following ONC. In addition to non-vascular tdTomato^+^ cells, it will be of interest in future studies to analyze the participation of tdTomato^+^ PCs in growing and matured functional vessels within the lesion over time. To assess neovascularization and PC coverage on longitudinal vessels, analysis of the complete optic nerve would be required (e.g., using light sheet microscopy). Furthermore, 60%–90% of the PDGFRβ^+^ cells in the lesion were also positive for the PC-derived tdTomato reporter. This is in line with CNS lesion studies reporting that 80%–90% of the PDGFRβ^+^ scar-forming cells originate from type A PCs (Dias et al., 2021).

Liu et al. (2021) recently investigated the cellular components of a fibrotic scar following ONC using the constitutive Col1a1-GFP transgenic mouse, established to label fibroblasts, including perivascular fibroblasts (Yata et al., 2003). By co-localization of Col1a1-GFP^+^ cells with PDGFRβ and aSMA, they concluded that the fibrotic scar in the ON is predominately formed by PCs (Liu et al., 2021). However, as PDGFRβ is also expressed in perivascular fibroblasts and aSMA identifies vSMCs but is absent in PCs (Trost et al., 2013), the Col1a1-GFP^+^ cells may represent perivascular fibroblasts rather than PCs. Using the same constitutive Col1a1-GFP reporter mouse in combination with a PC-specific inducible reporter mouse (NG2-CreER™-tdTomato), Soderblom et al. (2013) concluded that NG2-tdTomato^+^ PCs do not significantly contribute to the Col1a1-GFP^+^ cell population and are not a major source of the fibrotic scar upon SCI. In line, the participation of CNS fibroblasts, but not PCs or vSMCs, in fibrotic scar formation was demonstrated in an experimental autoimmune encephalomyelitis model using three different inducible reporter mouse lines (NG2, aSMA, and Col1a2) (Dorrier et al., 2021). Our findings, however, clearly indicate a participation of PC-derived tdTomato^+^ cells in fibrotic scar formation after ONC, constituting up to 90% of PDGFRβ^+^ scar-forming cells in the lesion. In line with our findings, the contribution of PC (subpopulations) to scar formation was reported in inducible lineage tracing models following SCI (Glast promoter) (Goritz et al., 2011) or vascular ischemia in the heart and brain (tbx18 promoter) (Pham et al., 2021).

Regarding the definition and discrimination of PCs and perivascular fibroblasts, it will be of highest interest to investigate the overlap of the cell populations targeted by e.g., the inducible NG2-CreER™ promoter, the inducible PDGFRβ-P2A-CreER^T2^ promoter and the constitutive Col1a1 promoter, to further deepen the understanding of the cellular components in the fibrotic scar. Importantly, as in constitutive Cre-lines reporter expression may be triggered upon injury and distinction of active and historical reporter expression is not possible, the use of inducible lineage tracing models is inevitable to investigate the origin of scar-forming (fibroblasts) cells in different types of (CNS) lesions. In this sense, the strength of this study lies in the use of an inducible mouse model, which allows unambiguous tracking of the originally labeled cells.

As the origin of collagen-producing cells following CNS injury is discussed controversially and as a subset of PCs is described to give rise to Col1-expressing cells (Goritz et al., 2011; Dias et al., 2021), besides the already described proliferation and migration of resident fibroblasts contributing to form a fibrotic scar (Soderblom et al., 2013; Dorrier et al., 2021), we aimed to investigate Col1a1 expression in tdTomato^+^ cells. As we failed to obtain specific collagen labeling in control skin tissue and lesioned ON tissue with the antibodies used, we applied the birefringence of collagen fibers and detected collagen deposition using polarization filters. Indeed, polarization signal was detected in close proximity to tdTomato^+^ cells, but it cannot be clearly concluded from this that tdTomato^+^ cells actually produced the collagen.

### 4.3 Glial activation after optic nerve injury

In addition to an accumulation of PDGFRβ^+^ cells in the ONC lesion, an immediate strong and persistent activation of the microglial marker Iba1 was detected in the lesion core up to 8 wpl. This is in line with other studies demonstrating a sustained high density of Iba1^+^ microglial cells at the crush site (Qu and Jakobs, 2013; Haga et al., 2016). Moreover, accumulation and activation of macrophagic/microglial cells at the lesion core after ONC has been described by others using ED-1 (Blaugrund et al., 1992; Frank and Wolburg, 1996; Podhajsky et al., 1997; Ohlsson et al., 2004; Chien et al., 2016), CD68 (Tonari et al., 2012), or CD11c^GFP^ mice (Heuss et al., 2018). Further analysis of Iba1-IR in the lesion core revealed a close proximity to tdTomato-IR and PDGFRb expression. However, further detailed studies are required to unequivocally assert co-expression in the same cell. Ki67 labeling was performed to analyze the proliferative state of the cells within the lesion. The majority of Ki67^+^ cells revealed a co-localization with Iba1 at 1 wpl, indicating a proliferative state of a subset of microglial cells at the time point investigated. This is in line with studies reporting proliferation of microglial cells after ONC, showing increased Ki67^+^ cells in the crushed ON 1 wpl (Heuss et al., 2018) or BrdU^+^ cells co-localizing with Iba1 (Qu and Jakobs, 2013). However, as Ki67-IR was almost absent in tdTomato^+^ cells 1 wpl, additional experiments are needed to unveil whether PC-derived scar-forming cells proliferate at different time points than the ones investigated here. Prolonged proliferation of PC-derived stromal cells is indicated by findings reported by Göritz et al. (2011), showing that 35% of PC-derived cells in the lesion are positive for Ki67 on day 5 post SC lesion. They further revealed that the number of PC-derived cells peaked 14 dpl, indicating proliferation of these cells up to 14 dpl. Consistent with this, we detected a significant increase of tdTomato+ cells from 1 wpl to 3 wpl, which remained constant at 8wpl, indicating cell proliferation/accumulation up to 3 wpl. Whether, in our lesion model, PC-derived stromal cells migrate and proliferate within the lesion or proliferate outside the lesion and invade the lesion needs to be clarified in future studies. In addition to microglial activation, GFAP^+^ astrocytes become reactive and form an (outer) glial scar, showing a clear demarcation from the inner fibrotic scar, which persisted up to 8 wpl, the latest time point investigated in our study. No co-localization was detected with tdTomato^+^ cells, clearly distinguishing astrocytes and tdTomato^+^ cells in the lesion upon ONC. This is in line with the findings of Liu et al. (2021), describing a clearly defined GFAP^+^ rim, surrounding the fibrotic component.

## 5 Conclusion

As the fibrotic scar is described to impede axonal regeneration, modulation of scar-forming cells is a promising strategy to improve regeneration. However, unveiling the cellular components of the fibrotic scar is one major prerequisite to develop treatment strategies. By using the inducible PDGFRβ-P2A-CreER^T2^-tdTomato lineage tracing reporter mouse, the present study reveals, for the first time, that following ONC, up to 90% of PDGFRβ^+^ scar-forming cells are derived from PCs. Although our study clearly indicates the importance of PCs in ON scar formation, thorough PC characterization and uniform definition are inevitable in order to specifically target PCs for the future development of anti-fibrotic therapies. Hence, further lineage tracing experiments following CNS injury, combined with single-cell transcriptomics analysis, may help to identify molecular signatures of PC (subpopulations) and their contribution to scar formation.

## Data Availability

The raw data supporting the conclusion of this article will be made available by the authors, without undue reservation.

## Abbreviations

CD31, cluster of differentiation 31; Col1a1, collagen type 1 alpha 1 chain; CreERT, Cre recombinase fused to modified estrogen receptor; Cspg4, chondroitin sulfate proteoglycan 4; DAPI, 4′, 6-diamidin-2-phenylindol; FoxD1, Forkhead box D1; GFAP, glial fibrillary acid protein; GFP, green fluorescent protein; GLAST, Slc1a3, sodium-dependent glutamate/aspartate transporter; Iba1, ionized calcium-binding adapter molecule 1; MBP, myelin basic protein; NG2, neural/glial antigen 2; Olig2, oligodendrocyte transcription factor 2; PDGFRβ, platelet-derived growth factor receptor beta; PLP, proteolipid protein; Sox10, SRY-box transcription factor 10; Tbx18, T-box transcription factor 18.
