# Supplementary material for: Pericyte-derived cells participate in optic nerve scar formation
Source: Front Physiol. 2023 Apr 18;14:1151495. doi: 10.3389/fphys.2023.1151495 (PMC10151493; doi:10.3389/fphys.2023.1151495)
Supplement: Supplementary file 1 [file Table1.DOCX]

**Figure Caption Supplemental Figure:**

**Supplemental Figure 1: PDGFRβ and Iba1 expression in the lesion.** (A) Iba1-IR (green) and (B) PDGFRβ-IR (red) analyzed in the lesion 8 wpl are in close proximity, (C) revealing overlapping IR at the cell margin/boundary (filled arrowheads).
