# Supplementary figures and images for: Pericyte-derived cells participate in optic nerve scar formation
Source: Front Physiol. 2023 Apr 18;14:1151495. doi: 10.3389/fphys.2023.1151495 (PMC10151493; doi:10.3389/fphys.2023.1151495)

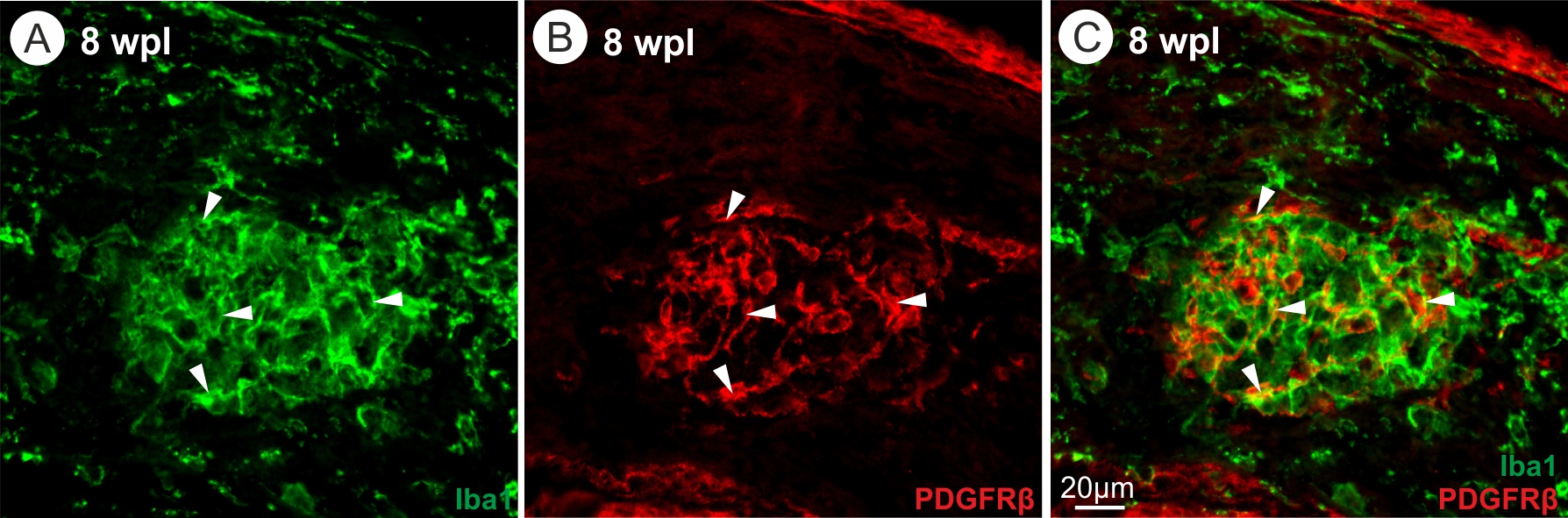

Supplement: Supplementary file 2 [file Image1.JPEG]
